# Functional characterization and architecture of recombinant yeast SWR1 histone exchange complex

**DOI:** 10.1093/nar/gkx414

**Published:** 2017-05-12

**Authors:** Chia-Liang Lin, Yuriy Chaban, David M. Rees, Elizabeth A. McCormack, Lorraine Ocloo, Dale B. Wigley

**Affiliations:** Section of Structural Biology, Department of Medicine, Imperial College London, London SW7 2AZ, UK

## Abstract

We have prepared recombinant fourteen subunit yeast SWR1 complex from insect cells using a modified MultiBac system. The 1.07 MDa recombinant protein complex has histone-exchange activity. Full exchange activity is realized with a single SWR1 complex bound to a nucleosome. We also prepared mutant complexes that lack a variety of subunits or combinations of subunits and these start to reveal roles for some of these subunits as well as indicating interactions between them in the full complex. Complexes containing a series of N-terminally and C-terminally truncated Swr1 subunits reveal further details about interactions between subunits as well as their binding sites on the Swr1 subunit. Finally, we present electron microscopy studies revealing the dynamic nature of the complex and a 21 Å resolution reconstruction of the intact complex provides details not apparent in previously reported structures, including a large central cavity of sufficient size to accommodate a nucleosome.

## INTRODUCTION

The SWR1 complex is a multi-subunit assembly found in yeast with a role in nucleosome histone exchange (1–3). The complex is assembled around a large Swr1 subunit that comprises several distinct domains including the HSA domain, that binds actin and Arps (4), and an ATPase domain that resembles that of DNA helicase/translocases (5). The protein appears to lack the canonical histone interacting domains (such as chromo and bromo domains) found in other nucleosome modifying complexes (6).

SWR1 complex has been purified from yeast using a tagged Swr1 subunit expressed endogenously (1,3,7). The complex is able to catalyse the stepwise and ATP-dependent exchange of canonical H2A/H2B histone dimers with Htz1/H2B dimers (7). Despite containing ATPase domains resembling those of other ATP-dependent nucleosome-sliding complexes that are characteristic of DNA translocases (5) such as Ino80 (8), the SWR1 complex is unable to mobilize nucleosomes under similar conditions for which nucleosome sliding by Ino80 can be observed (3,8).

Using recombinant expression in insect cells we have managed to prepare large quantities of a fourteen subunit SWR1 complex for biochemical and structural analysis. We have also developed fluorescence-based assays to characterize histone exchange as well as nucleosome binding and ATPase activities of this complex. The complex has catalytic histone exchange activity that is comparable to full complex isolated from yeast (9). We have prepared complexes that are deficient in selected subunits to shed light on the roles of these subunits in histone exchange. N-terminal truncations of the Swr1 subunit reveal how the other subunits associate with the main Swr1 scaffold as well as providing further information about their interactions. C-terminal truncations uncover an interaction between a second actin and the Swc5 subunit. Finally, we present electron microscopy data to analyse the dynamic conformational nature of the complex. A 21 Å resolution reconstruction of one of these conformational states is similar in overall shape to that published previously for a crosslinked complex (10), but reveals additional details about the complex that were not evident previously.

## MATERIALS AND METHODS

### Cloning and purification of yeast SWR1 complexes

Genes for a fourteen subunit yeast SWR1 complex comprising Swr1 (residues 1–1514 with an N-terminal 8-histidine and C-terminal twin Strep-tag^®^), actin, Arp4, Arp6, Bdf1, Yaf9, Swc2, Swc3, Swc4, Swc5, Swc6, Swc7, Rvb1 and Rvb2 were synthesized with codon bias for expression in insect cells (Genscript). Genes were cloned into transposition-compatible vectors using the MultiBac system (11). Swr1 N- and C-terminal truncation mutants were constructed by In-Fusion cloning (Clontech). The constructs used to express SWR1 sub-complex 1 (SC1) containing Swr1 residues 1–681 (NTD) or Swr1 residues (1–681)-(GGGS)_2_-(1360–1514) (ΔATPase) were generated by In-Fusion cloning. The actin binding site deletion mutants, ΔA1 (missing residues 370–409), ΔA2 (missing residues 410–459), ΔA1A2 (missing residues 370–459) were also constructed by In-Fusion cloning. Genes were omitted as required for the production of mutant deletion complexes.

Complexes were expressed in BTI-TN-5B1-4 (High Five) insect cells at 27°C for 72 h post-infection in Insect-XPRESS™ Protein-free Insect Cell Medium with l-glutamine (Lonza). Cells were harvested by centrifugation at 2000 rpm for 20 min at 4°C. Purification used a two-step purification protocol. Following lysis by sonication in Buffer A [50 mM Tris (pH 8.0), 500 mM NaCl, 5% glycerol, 1 mM DTT, 1 mM EDTA, 2 mM MgCl_2_, 2 mM benzamidine–HCl, 2 mM 6-aminocaproic acid] supplemented with 5 μl Benzonase^®^ nuclease (Sigma-Aldrich) and one cOmplete Protease Inhibitor Tablet (Roche) per liter of initial cell culture volume, lysates were clarified by centrifugation at 20 000 rpm for 1 h at 4°C and filtered through a 0.45 μm filter before being loaded onto a StrepTactin HP column (GE Healthcare). The column was washed in Buffer A and eluted with Buffer B [25 mM HEPES (pH 7.5), 100 mM KCl, 10% glycerol, 1 mM DTT, 1 mM EDTA, 2 mM MgCl_2_, 0.01% NP-40] supplemented with 2.5 mM desthiobiotin. The eluted protein was applied to a HiTrap Q HP column and eluted with a gradient of 0–1 M KCl. The protein was concentrated to around 10 μM in storage buffer B and flash frozen in liquid nitrogen.

### Preparation of nucleosomes

All experiments utilized yeast nucleosomes reconstituted from histones expressed in *Escherichia coli* and assembled on DNA fragments based on the Widom 601 positioning sequence (12). Yeast H2A and H2B or Htz1 and H2B were co-expressed in *E. coli*. Cells were lysed by sonication in Lysis Buffer (20 mM Tris pH 7.5, 400 mM NaCl, 0.1 mM EDTA, 1 mM TCEP plus 2 Roche Protease Inhibitors tablets per 100 ml). H2A/H2B or Htz1/H2B dimers were purified as soluble proteins by HiTrap Q FF, HiTrap Heparin HP in Buffer A (20 mM Tris pH 7.5, 400 mM NaCl, 1 mM EDTA, 1 mM TCEP) and eluted off the Heparin column by a salt gradient by mixing with Buffer B (20 mM Tris pH 7.5, 2 M NaCl, 1 mM EDTA, 1 mM TCEP), followed by gel filtration on Superdex S200 in Buffer A. Dimers were labelled with Alexa Fluor 555 C_2_ maleimide (AF555, Life Technologies) on H2A^K119C^ or Htz1^K125C^ and re-purified by gel filtration on Superdex S200 in Buffer A.

Yeast octamers were co-expressed in *E. coli*, lysed in Lysis Buffer and purified as soluble octamers on HiTrap Heparin HP in Buffer A and eluted with a salt gradient by mixing with Buffer B, followed by Superdex S200 in Buffer B. Octamers were labelled with AF555 on H2A^K119C^ or Htz1^K125C^ and re-purified by gel filtration on Superdex S200 in Buffer B.

Yeast nucleosomes were reconstituted from octamers and DNA by salt gradient dialysis in several steps from 2 to 0.2 M NaCl. The DNA fragment used was a 153 bp DNA fragment based on a 167 bp Widom fragment provided by Daniela Rhodes (13) that was further digested with HinfI. End-positioned nucleosomes with a 101 bp overhang or an Alexa Fluor 647 C_2_ maleimide (AF647, Life Technologies) label on the short DNA tail were prepared as previously described (14).

### ATPase assays

ATPase activity of the complex was measured by a coupled assay that measures ADP release as described previously (15) but utilizing NADH fluorescence rather than absorbance to increase sensitivity (14). A final concentration of 100 mM NADH, 0.5 mM phosphoenolpyruvate, 100 U/ml pyruvate kinase (Sigma), 20 U/ml lactate dehydrogenase (Sigma) were used in all reactions in a final volume of 50 μl. Typical reactions were conducted using 20 nM purified SWR1 complex, 200 nM yH2A-containing end-positioned nucleosome with a 101 bp overhang and 1 mM ATP. ATP solutions were made with a ratio of 1:2 ATP:TRIS base. Reactions were conducted by mixing all components immediately prior to transferring to an Optiplate-384 Black Opaque 384-well microplate (Perkin Elmer) that had been pre-incubated at 30°C. Reactions were initiated with the injection of ATP using the built-in reagent injectors, which were pre-filled with 10× ATP solution. Reactions were monitored fluorescently using an excitation of 335 nm and an emission of 469 nm at 30°C with a CLARIOstar^®^ microplate reader (BMG Labtech). All reaction rates were determined using the maximum initial linear rate and reaction kinetics were analysed assuming a Michaelis–Menten model. Where it is used as a comparison, wild type complex was assayed on the same day with the same assay reagents for better direct comparison. The rates in different figures therefore differ from one another.

### Histone exchange assays

For gel-based assays, end-positioned nucleosomes with a 101 bp overhang and Alexafluor AF555 labelled histone dimer were prepared as described above. For a typical assay, frozen stocks of SWR1 complex were rapidly thawed and diluted to 10× working concentration (0.5 μM) in Assay Buffer [25 mM HEPES (pH 7.5), 100 mM KCl, 10% glycerol, 1 mM DTT, 1 mM EDTA, 2 mM MgCl_2_, 0.01% NP-40] and then pre-incubated at 30°C with yeast H2A- or Htz1-containing nucleosomes and yeast H2A–H2B or Htz1–H2B dimer labelled with Alexafluor AF555 at a final concentration of 200 and 400 nM, respectively. Reactions were started by adding 1 mM ATP and terminated by addition of EDTA (10 mM) and salmon sperm DNA (1 μg). Reaction products were resolved by native gel electrophoresis using 6% acrylamide–TBE gels, run in 0.5× TBE buffer at 90 V for 90 min at 4°C. Gels were visualized and digitized for quantification using a Bio-Rad ChemiDoc MP system.

FRET-based microtitre assays for measuring histone exchange by recombinant SWR1 complexes were performed on a CLARIOstar^®^ microplate reader (BMG Labtech) in a final volume of 50 μl in OptiPlate-384 Black Opaque 384-well microplates (Perkin Elmer). Yeast H2A-containing, end-positioned nucleosomes (with a 101 bp overhang and labelled on histone H2A with AlexaFluor 555 (AF555) (Life Technologies) and AlexaFluor 647 (AF647) (Life Technologies) on the 5΄ DNA end closest to the histone core) and yeast Htz1–H2B dimer were prepared in Assay Buffer [25 mM HEPES (pH 7.5), 100 mM KCl, 10% glycerol, 1 mM DTT, 1 mM EDTA, 2 mM MgCl_2_, 0.01% NP-40]. For a standard assay, the final working concentrations of SWR1 complex, nucleosome and histone dimer were 50, 200 and 400 nM, respectively. Reactions were initiated with the injection of ATP to a final concentration of 1 mM using the built-in reagent injectors, which were pre-filled with 10× ATP solution (10 mM ATP). The exchange reaction was monitored via the decrease in fluorescence of AF647 (excitation at 535 nm and emission at 680 nm), with readings taken every 60 s.

### Nucleosome binding assays

Native agarose gel electrophoresis was used to determine binding constants for nucleosome binding to SWR1 complexes. SWR1 complex-nucleosome interactions were measured using yeast H2A-containing, end-positioned nucleosomes (with a 101 bp overhang and labelled on histone H2A with AlexaFluor 555 (AF555) (Life Technologies) and AlexaFluor 647 (AF647) (Life Technologies) on the 5΄ DNA end closest to the histone core). For each set of measurements, a 2-fold serial dilution of wild type SWR1 complex, or mutant variants, was prepared at 2× final concentration (starting at 4 μM) in Assay Buffer [25 mM HEPES (pH 7.5), 100 mM KCl, 10% glycerol, 1 mM DTT, 1 mM EDTA, 2 mM MgCl_2_, 0.01% NP-40] before mixing with an equal volume of 10 nM nucleosome stock solution (also in Assay Buffer), to yield a final nucleosome concentration of 5 nM. Samples were given 15 min to equilibrate before being loaded into 0.8% native agarose gel and running the gel at a constant voltage of 30 V for 1 h at room temperature. Gels were visualized and digitized for quantification using a Bio-Rad ChemiDoc MP system. The *K*_d_ value was estimated using GraphPad Prism 6.

### Sample preparation for electron microscopy

3 μl of protein sample was applied to a freshly glow discharged continuous carbon film grid (carbon film over Quantifoil 2/2 holey carbon grid) and incubated for 20 s, washed twice with buffer and stained with two 6 μl drops of 2% uranyl acetate. Data were recorded on an F20 microscope (FEI) operated at 200 kV at nominal magnification of 29 000. A Tietz F415 CCD camera was used for data collection. The pixel size at the level of the specimen was 2.75 Å/pixel. In order to improve signal, three micrographs of each area were recorded, translationally aligned to account for drift, and averaged using the SPIDER software package (16). The dose was 10–15 electrons/Å^2^ per micrograph with a total accumulated dose of 30–45 electrons/Å^2^ (Supplementary Figure S5).

### EM data analysis

The defocus of the micrographs was estimated using EMAN (17). CTF correction by phase flipping was performed in SPIDER (16). In order to minimize variations due to stain level and the amount of defocus, amplitudes of all CTF corrected micrographs were normalized. The reference for amplitude normalization was a smoothed average of amplitudes from micrographs at different defocuses. To remove uneven staining artifacts, amplitudes below a frequency of 0.01 were suppressed (Supplementary Figure S5). A total of 21 108 particles were manually selected from 84 summed micrographs using BOXER in the EMAN software package (17). Initial image analysis was performed with IMAGIC (18). Particles were centered using a rotationally averaged sum of all particles and subjected to Multivariance Statistical Analysis (MSA) and classification. Representative classes were selected as references for particle alignment. Criteria for class selection were good signal-to-noise (SNR) ratio and ‘uniqueness’ of view: only the best class-average was selected from similar looking classes, while classes with lower SNR were allowed for unique views. Aligned data were subjected to another round of MSA and classification to obtain better class averages.

### Generation of initial EM models

Examination of the initial classes suggested conformational flexibility of the complex. In order to obtain models reflecting different conformations, we selected classes containing clearly identifiable side views of the Rvb1/2 ring. A bright line representing the side view of electron dense Rvb1/2 AAA+ ATPase ring was used as a criterion for class selection and subsequent alignment (Supplementary Figure S5). Measurements of the length of the average in classes indicated a variability in length of ∼100 Å between the extremes. Classes were divided into five groups, with each group representing ∼20 Å increase in length. Initial models were generated from each group using Angular Reconstitution in IMAGIC (18).

### Model refinement

Initial models were refined in RELION (19). 3D classification was used to sort non-side view particles between different models and split the full data set into subsets containing 4000–6000 particles. Models for each subset were refined in RELION reaching resolutions of 24–27 Å at this stage when estimated using the gold standard Fourier Shell Correlation method (20) (Supplementary Figure S6). The most compact SWR1 model had the best overall resolution and was selected for further refinement, finally reaching a resolution of 21 Å (Supplementary Figure S7). Local resolution for this reconstruction was estimated using ResMap (21) and was in a range of 14–24 Å (Supplementary Figure S7). The final reconstruction has been deposited at the EM database with ID code EMD-3607.

### Docking of atomic coordinates

The best quality model, representing the most compact conformation of SWR1, was chosen for docking of Rvb1/2 atomic coordinates (PDB accession code: 4WVY (22)). The Rvb1/2 ring density could be distinguished clearly from the rest of the SWR1 complex (Figure 6A). Initial docking was performed using the ‘Fit in Map’ option in UCSF Chimera (23). While the fit of Domains I and III (DI, DIII) of the Rvb1/2 heterohexamer was satisfactory, it was clear that five out of six subunits of the Rvb1/2 ring in the SWR1 model are in an extended conformation of Domain II (DII) which is best described by the Rvb1 subunit conformation in 4WVY. The sixth monomer was less extended, with DII apparently in an intermediate conformation between the corresponding domain in the Rvb1 and Rvb2 subunits in the 4WVY model. The end of DII contains a structurally conserved OB domain, while the preceding DII linker is flexible (22). Thus, to obtain a better fit for the Rvb1 ring we performed a rigid body fit of the OB domain into the SWR1 density using the Phenix software (24) along with the other subunits. The cross-correlation between the OB domains and the EM map density increased from 0.87 to 0.92 after this procedure. Figure 6B shows the fit of the Rvb1/2 model into the SWR1 map. The docked model was used to separate density for the Rvb1/2 ring from the rest of the SWR1 complex using the Chimera integrated Segger package (25) (Figure 6C).

## RESULTS

### Expression of recombinant ySWR1 complex in insect cells

Previous work on yeast SWR1 (ySWR1) complexes has used protein extracted from cells using a tagged Swr1 subunit (3,7). However, SWR1 complex expression is at low levels in yeast cells so we decided to circumvent this by expressing recombinant complex in insect cells. We used the MultiBac expression system (11) to produce the complex in insect cells that comprised fourteen subunits (Swr1, actin, Arp4, Arp6, Yaf9, Bdf1, Swc2, Swc3, Swc4, Swc5, Swc6, Swc7, Rvb1 and Rvb2) (Supplementary Figure S1A). Identities of the bands on the SDS gel were confirmed by mass spectrometry, together with a variety of deletion complexes (see below).

### Nucleosome histone exchange assays

**Figure 1.**
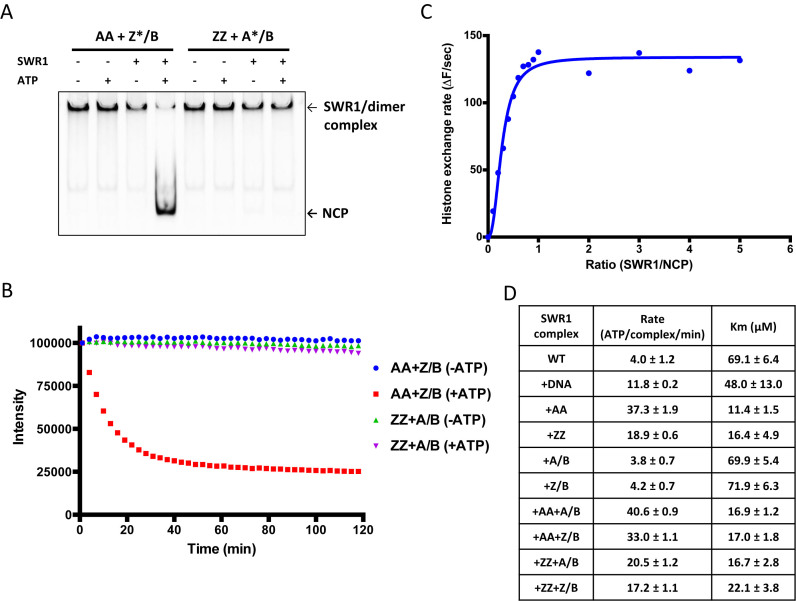
Characterisation of the wild type recombinant SWR1 complex. (**A**) Fluorescence scan of gel-based histone exchange assay performed as described in the Methods section. AA and ZZ denotes canonical nucleosomes containing H2A or Htz1 histones, respectively, Z/B and A/B denotes Htz1/H2B or H2A/H2B dimers, respectively. Fluorescently-labelled dimers become incorporated into nucleosomes only in the presence of SWR1 complex and ATP, (**B**) FRET-based histone exchange assay. When fluorescent-labelled H2A/H2B dimer is exchanged with unlabelled Htz1/H2B dimer, the FRET signal with the bound DNA is lost, (**C**) titration of enzyme with a fixed amount of nucleosome at high concentration (200 nM) reveals maximal activity at a ratio of one SWR1 per nucleosome, (**D**) ATPase data with a variety of nucleosome and dimer combinations. Where wild type ATPase rates are used for comparison in later figures, these were measured on the same day with the same reagents for better comparison with mutant complexes.

SWR1 complex has been shown to have nucleosome histone exchange activity (3,7). We developed a gel-based assay that monitors incorporation of a fluorescently-labeled Htz1/H2B dimer into nucleosomes. This showed that our recombinant yeast SWR1 complex also catalyses histone exchange (Figure 1A and Supplementary Figure S3A) at a rate comparable to that reported for the tagged endogenous yeast complex (9). Previous assays in (9) at a ratio of 1:5 SWR1:nucleosome showed 70% completion in 60 min. For our assay, at a ratio of 1:4 SWR1:nucleosome showed 90% completion in 60 min. This activity is also catalytic and our enzyme is capable of catalyzing at least eight turnovers.

We also developed a FRET-based assay to use in a microtitre plate reader (Figure 1B and Supplementary Figure S3D). The results for histone exchange obtained with this assay are similar to those from the gel-based assay but with continuous time point measurements. Consequently, we used this assay for subsequent analysis.

### A SWR1 complex monomer is sufficient to catalyse histone exchange

Assays conducted at high nucleosome concentrations show that the activity peaks at a ratio of one SWR1 complex per nucleosome (Figure 1C). It has been shown that both H2A/H2B dimers can be exchanged by SWR1 (7). Our observation that a single complex is required for peak activity precludes a mechanism by which two complexes could bind simultaneously to a single nucleosome to exchange both dimers in a processive manner. However, our current data cannot determine whether a single complex acts processively or distributively to effect exchange of the two dimers.

### ATPase activity

ATPase activity of the complex was measured by a coupled assay that measures phosphate release as described previously but utilising NADH fluorescence rather than absorbance to increase sensitivity (14) (Figure 1D). The rates were similar to values reported previously (7,9). We used nucleosomes containing either H2A or Htz1 but the ATPase activity was stimulated maximally by those containing H2A. Unlike the endogenous SWR1 complex (7), we do not observe hyperstimulation of ATPase by Htz1/H2B dimers.

### Properties of mutant complexes lacking subunits

**Figure 2.**
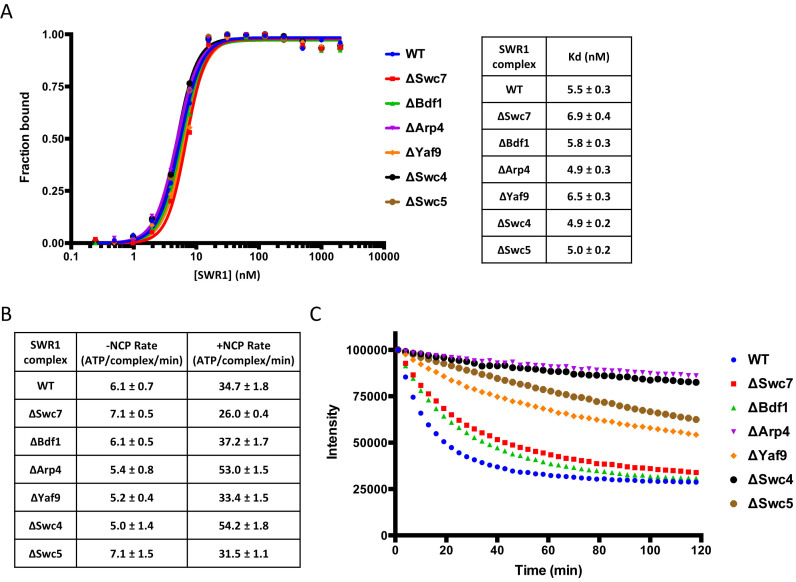
Analysis of subunit deletion complexes. (**A**) Nucleosome binding affinities. Raw data from gel shift assays are presented in Supplementary Figure S4. The gels were scanned and plotted (left) with tabulated *K*_d_ values (right), (**B**) ATPase rates, (**C**) Histone exchange activity.

Having established a set of assays to monitor these basic activities of the complex, we then prepared a series of complexes that lacked different subunits (Supplementary Figure S1A). The deletion of subunits had a variety of consequences on the complex giving clues about interactions between proteins in the complex. The deletion studies also provided us with several different complexes that were assessed for exchange activity as well as ATPase and nucleosome binding (Figure 2).

Deletion of actin resulted in a complex that still contained that subunit because it picks up the closely related insect cell actin in its place (Supplementary Figure S1A). We have also seen this for recombinant human Ino80 complex (14). Consequently, this complex was not characterised further. Several single subunit deletions resulted in the (often partial) loss of multiple subunits, as seen previously for endogenous ySWR1 (26,27). We repeated some of the previously reported deletions as well as making others that were not possible in yeast because the genes are essential. Single protein deletions were possible for Bdf1, Swc5 and Swc7. Deletion of either Bdf1 or Swc7 gave only modest impairment of histone exchange activity and the binding affinity of these complexes for nucleosomes was unaffected by the loss of these subunits (Figure 2). By contrast, deletion of Swc5 resulted in a significant loss of exchange activity although the ATPase rate and affinity of the complex for nucleosomes were essentially the same as the full complex (Figure 2). This demonstrates a role for Swc5 in coupling ATPase to histone exchange.

Deletion of Yaf9 resulted in loss of both Yaf9 and Bdf1 indicating an interaction between them although loss of Bdf1 does not result in a loss of Yaf9 (Supplementary Figure S1A). The resulting complex, lacking Yaf9 and Bdf1, shows essentially unaltered nucleosome affinity but is severely impaired in exchange activity despite retaining normal ATPase (Figure 2). As for the Swc5 deletion, this again suggests a role for Yaf9 in coupling ATP hydrolysis to histone exchange.

Arp4 is an essential gene in yeast (28). Consequently, previous studies utilised a degron strain to reduce the levels of Arp4 to study the role of this subunit in endogenously produced SWR1 complex. Although this produced a complex that still retained partial occupancy with Arp4, the activity was greatly reduced (27). Our recombinant expression system allowed us to prepare an Arp4 deletion complex that completely lacks Arp4 (Supplementary Figure S1A). In common with the previous studies, loss of Arp4 also resulted in partial loss of several other subunits (actin, Bdf1, Swc4 and Yaf9) (Supplementary Figure S1A) suggesting a network of interactions between these subunits. Perhaps understandably, given how many subunits are lost, this complex showed no histone exchange activity although its nucleosome affinity was unaltered (Figure 2). Interestingly, the nucleosome-stimulated ATPase rate of the Arp4 deletion complex was actually slightly higher than that of the wild type showing this was now uncoupled from histone exchange (Figure 2B).

Finally, we were able to create a Swc4 deletion complex that was not possible with endogenously prepared material (26). In addition to the loss of Swc4, we also observe complete loss of the Bdf1 and Yaf9 subunits as well as partial loss of Arp4 (Supplementary Figure S1A). The complex lacks exchange activity but has similar affinity for nucleosomes as wild type (Figure 2). As for the Arp4 deletion complex, which lacks similar subunits, the nucleosome-stimulated ATPase rate was slightly enhanced over wild type showing an uncoupling of histone exchange from ATP hydrolysis.

### Complexes with N-terminal truncations of the Swr1 subunit

In order to understand more about interactions between subunits within the complex, we next prepared a series of complexes containing N-terminally truncated Swr1 (Supplementary Figure S1B). These studies resulted in complexes that lacked an increasing number of subunits and provided useful information about interactions between Swr1 and these subunits. Removal of the first 210 residues of Swr1 resulted only in the loss of the Swc7 subunit showing an interaction with this region as shown previously by deletion studies (27). Loss of a further 54 residues resulted in the additional loss of the Bdf1 subunit suggesting it interacts directly with a region of Swr1 between residues 211 and 264. Previous studies (26) had suggested that this subunit interacted only with Yaf9 on the basis of subunit deletion experiments similar to those we carried out above. Our truncation data show this is probably not the case. Further truncation of Swr1 to residue 299 had no additional effect on the subunit composition of the complex. However, deletion of 339 residues had a dramatic effect, with additional complete loss of Swc4 and Yaf9 as well as partial loss of Arp4, suggesting binding sites for the Swc4 and Yaf9 subunits are located between residues 300 and 339 of Swr1. Given the results of subunit deletions described above, this strongly suggests that a binding site for Swc4 lies in this region. Truncation by an additional 30 residues (to residue 369) results in complete loss of Arp4. Further truncation (to residue 409) resulted in partial loss of the actin band. These results are consistent with the binding sites observed in a recently reported crystal structure of this region of Swr1 complexed with actin and Arp4 (29). However, we note that in other, less truncated, complexes (Supplementary Figure S1B), the actin band is stronger than those adjacent on the SDS gel (Swc5 and Arp6) but, in the complex truncated at residue 410, the actin band is now of a similar intensity to that of Swc5 and Arp6. This suggests there are two actin subunits with separate binding sites. To investigate this further, we made additional truncations of Swr1. Removal of another 50 residues resulted in complete loss of actin (Supplementary Figure S1B) suggesting the binding site for the second actin molecule lies between residues 410 and 459. Interestingly, the complete loss of the actin band was accompanied by complete loss of Swc5 although there was no loss of Swc5 associated with the initial loss of actin. Further truncation to residue 662 of Swr1 (Supplementary Figure S1B) showed no further loss of subunits.

**Figure 3.**
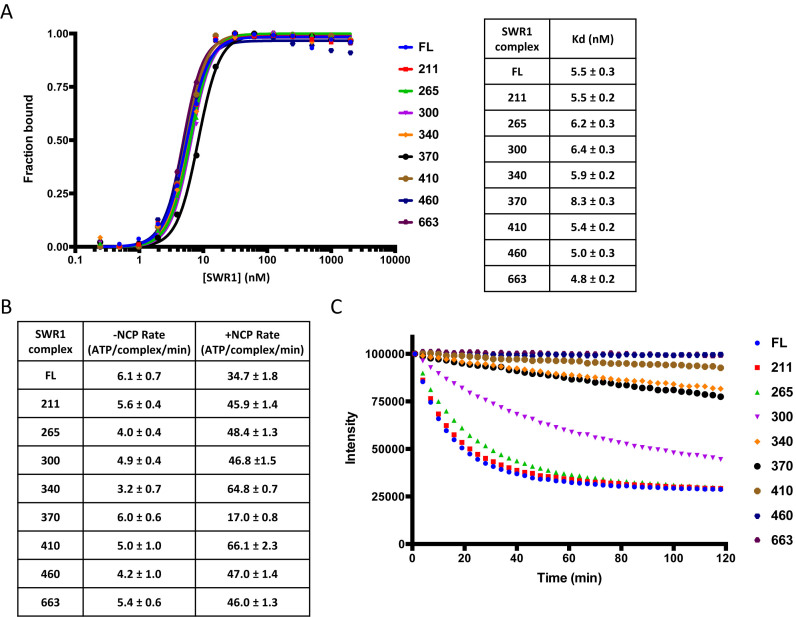
Biochemical analysis of Swr1 N-terminal truncation complexes. (**A**) Nucleosome binding affinities. Data from gel shift assays (left) and tabulated Kd values (right), (**B**) ATPase rates, (**C**) Histone exchange activity.

The activities of these truncation complexes were also analysed (Figure 3). Truncation to residue 264 saw little change in ATPase or exchange activities, or in affinity for nucleosomes. This is in line with the subunit deletion complexes that also lacked the Bdf1 and Swc7 subunits (Figure 2). Further truncation to residue 299 saw a reduction in histone exchange but little change in ATPase or nucleosome affinity. This is particularly interesting because the subunit composition does not alter as a consequence of this increased truncation (Supplementary Figure S1B), indicating a role for this region of Swr1 in coupling ATPase to histone exchange. Further truncations begin to induce significant loss of subunits with the expected loss in histone exchange activity (Figure 3C and Supplementary Figure S1B). However, the nucleosome affinity and ATPase activities are not affected significantly, again consistent with the subunit deletion complexes (Figure 2).

### Complexes with C-terminal truncations of the Swr1 subunit

**Figure 4.**
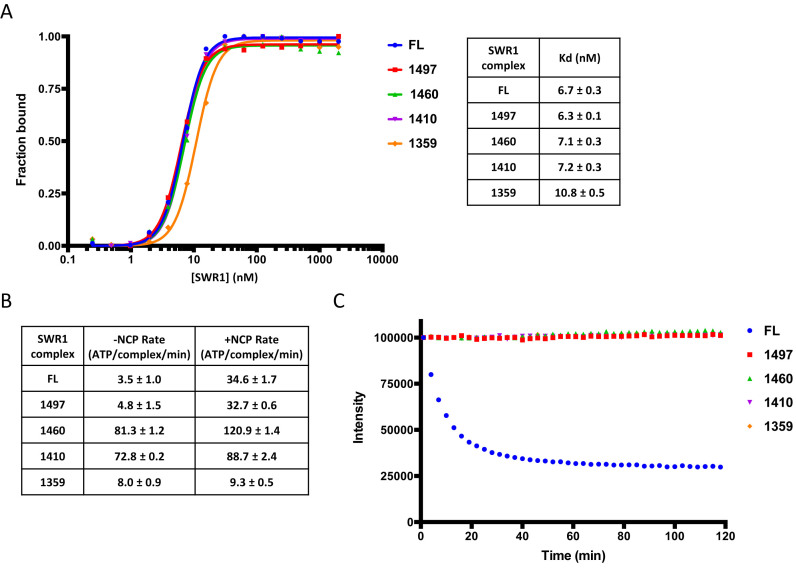
C-terminal truncation complexes of Swr1. An SDS gel of Swr1 C-terminal deletion complexes is presented in Supplementary Figure S2A. (**A**) Nucleosome binding affinities. Data from gel shift assays (left) tabulated *K*_d_ values (right), (**B**) ATPase rates, (**C**) Histone exchange activity.

We next prepared a series of complexes containing C-terminally truncated Swr1 subunit (Supplementary Figure S2A). Initial removal of just 17 residues (truncated at residue 1497) produced a complex that had almost completely lost the Swc5 subunit but also a noticeable loss of the actin band (Supplementary Figure S2A). This complex showed a complete loss of histone exchange activity (Figure 4). However, the nucleosome affinity and ATPase activity were unaffected (Figure 4A and B). Further truncations showed similar intensities for protein bands until truncation to 1359 at which point there seems to be some loss of Swc6 (and probably Arp6 since these form a pair (26). However, for truncations at residues 1410 and 1460 the ATPase activity was raised significantly above that of the full length complex and the regulation induced by nucleosome binding has been reduced substantially (Figure 4B). Further truncation to 1359 resulted in loss of nucleosome stimulated ATPase even though nucleosome binding was barely affected (Figure 4B). Consequently, this suggests a role for the C-terminal Swr1 residues coupling ATPase to exchange. The loss of coupling in the truncation mutants is not simply a consequence of losing Swc5 because deletion of this subunit in the context of a full length Swr1 subunit retains residual exchange activity, albeit reduced compared to wild type (Figure 2C).

Given the surprising role of the C-terminal 17 residues in binding Swc5 and an actin molecule, we decided to delete the ATPase domains of the Swr1 subunit and then re-attach the C-terminal regions to the N-terminal domain (NTD) via a flexible linker to see which subunits could be retained in addition to those associated with the NTD alone (Supplementary Figure S2B). The fusion of these 155 C-terminal residues to the NTD allowed association of the Swc5 along with an increase in the intensity of the actin band. This increase in intensity suggests association of a second actin, consistent with observations described above regarding the stepwise loss of the actin band in N-terminal and C-terminal truncation experiments.

### SWR1 complex contains two actin molecules bound at distinct sites

**Figure 5.**
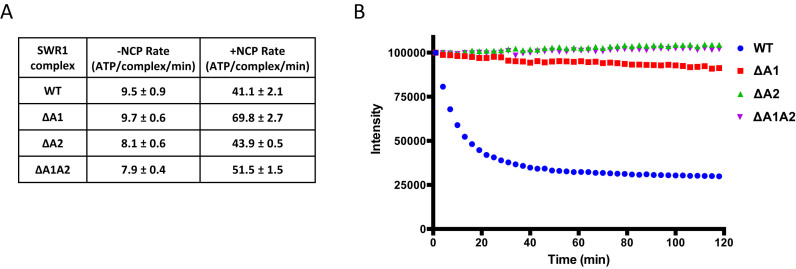
Internal deletion complexes. SDS gels of the NTD truncation complex, the DATPase domain Swr1 complex and the Swr1 actin-binding site deletion complexes are shown in Supplementary Figure S2. (**A**) ATPase rates for actin-binding site deletion complexes, (**B**) histone exchange activity of actin-binding site deletion complexes.

To formally confirm the existence of the two separate actin molecules, we selectively deleted the regions of the Swr1 subunit that correspond to each of the binding sites suggested by the N-terminal truncation experiments (Supplementary Figure S2C). Consistent with the results described above, we observed partial loss of actin associated with each deletion and complete loss when both were deleted. Furthermore, selective deletion of the second actin molecule also resulted in loss of Swc5, confirming our assignments of interactions predicted from the experiments above. ATPase activities and ATP-stimulation by nucleosomes were the same as for the full length complex (Figure 5A). However, all complexes were severely defective in histone exchange (Figure 5B), presumably due to the loss of Swc5, although the DA1 complex, to which Swc5 remains bound, retained a residual level of activity.

### Electron microscopy reconstructions

**Figure 6.**
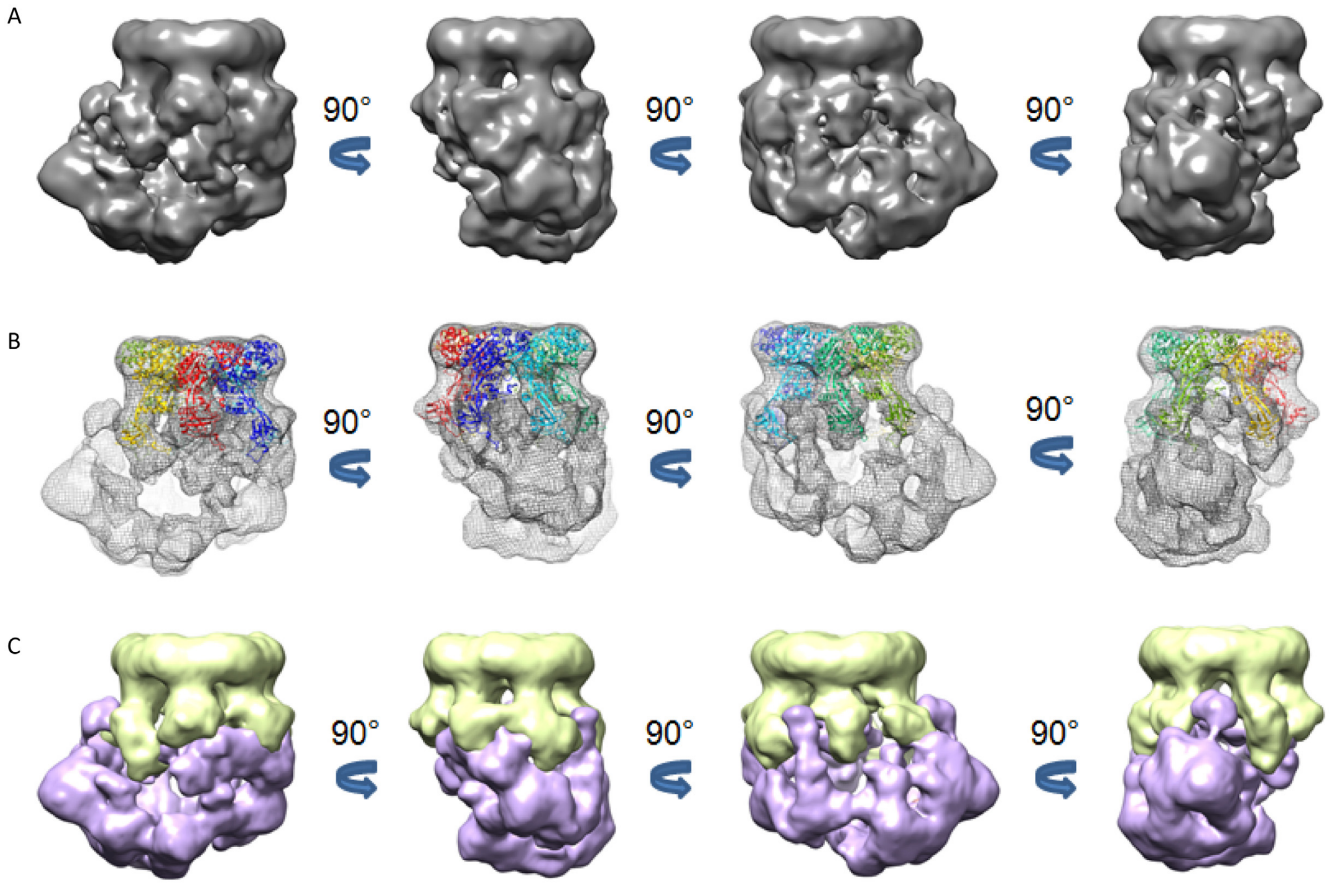
Negative stain electron microscopy reconstructions at 21Å resolution. (**A**) views of the final model rotated to different views as indicated, (**B**) Electron density of the model with Rvb1/2 hexamer coordinates docked as described in the Materials and Methods, (**C**) Segmentation of electron density with Rvb1/2 complex (lime green) and the remainder of the density in purple.

Previous studies have determined low resolution structures for SWR1 and SWR1:nucleosome complexes (10,30). These studies confirmed that the stoichiometry of the Rvb1/2 proteins in the complex was a single heterohexamer. While these structures are informative, the authors found it necessary to crosslink the complex with glutaraldehyde in order to stabilize the complex in a homogeneous state suitable for EM studies. By contrast, we have been able to determine conditions for negative stain that permit analysis of the complex without crosslinking (Figure 6), although we still observe substantial conformational heterogeneity. By splitting the particles into a number of classes with different average conformations (Supplementary Figures S3 and S4) we have been able to obtain five reconstructions, the best of these (Class 1 in Supplementary Figure S4) being at a resolution of 21 Å (Figure 6A, Supplementary Figures S5–S8). These studies reveal an overall structure that has considerable similarity with that reported previously. However, our structure is of a slightly more open complex that reveals a cavity within the complex of a suitable size to accommodate a nucleosome (Supplementary Figure S8). Crosslinking the complex with glutaraldehyde may have stabilised a closed conformation of the complex that, at low resolution, appears to be solid and in which the cavity is less evident. The crystal structure of a Rvb1/2 heterohexamer could be readily fitted into the density with minimal rigid body fitting (Figure 6B). This allowed us to assign the Rvb1/2 hexamer density (Figure 6C) and determine where the additional parts of the complex are located although the resolution is insufficient to assign subunit identities to the remaining density.

## DISCUSSION

Using the MultiBac expression system we were able to co-express the full fourteen subunit yeast SWR1 complex in insect cells and make active recombinant complex in much higher yield than in yeast cells, allowing us to prepare milligram quantities of purified complex. This will facilitate on-going structural and functional studies. This complex was as active in histone exchange as endogenously prepared ySWR1 (9). We developed a fluorescence-based gel assay to monitor exchange and also a FRET-based assay for continuous measurement in a microtitre plate reader. These will facilitate our future studies. The assays reveal that although the reaction rate for exchange is rather slow, in common with the endogenous complex, it is catalytic and can complete exchange of both H2A/H2B dimers in nucleosomes with Htz1/H2B, although we cannot yet determine whether these steps are processive or require re-association of the enzyme complex with nucleosomes. The large amounts of recombinant protein we are able to prepare allowed us to carry out histone exchange at high protein concentrations to analyse the stoichiometry required for activity and we find that a single SWR1 complex is sufficient to effect dimer exchange. This is important because some remodelling complexes have been shown to act as dimers (31) and given that two exchange reactions can be catalysed per nucleosome, it reveals that this is not carried by separate complexes simultaneously. It therefore seems most likely that the reaction requires disassociation and rebinding to effect the second exchange, although a processive mechanism cannot be discounted.

The subunit deletion complexes we prepared have shed some light on the function of the various subunits within this functional complex. In general, the results of our experiments were very similar to studies of endogenous protein prepared from yeast (7,9,26,27) although we were able to make complete knockouts of some proteins that are essential in yeast and therefore could not be prepared endogenously. Perhaps the most important finding was that the subunit deletions had little effect of the ATPase or nucleosome binding affinities of the complexes although several had reduced histone exchange activity. These data point to a role for these subunits in coupling the ATPase activity to histone exchange by an, as yet, undetermined mechanism.

**Figure 7.**
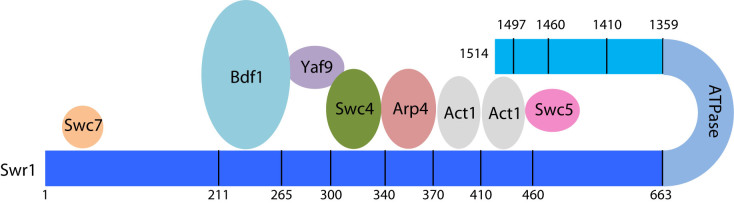
Cartoon summary of interactions between subunits and the Swr1 subunit. Direct and indirect interactions are based on the simplest interpretation of our data although additional interactions cannot be ruled out.

Previous studies have shown that the Swr1 subunit acts as a scaffold upon which many of the other subunits assemble and make direct interactions. Our N-terminal truncations of Swr1 complement the deletion studies, provide new information about interactions of a number of subunits with the N-terminal region of Swr1, and locates their binding sites. These interactions are summarised in the cartoon in Figure 7. Subunit deletion studies, presented here and by others (26,27), are generally in good agreement although we do see some significant differences. We identify a direct interaction between Bdf1 and the Swr1 subunit. Also, the binding of Bdf1 appears to be independent of Swc7 binding since both individual subunit deletions are clean with loss of the deleted subunit but retention of all others in the complex. The N-terminal truncations also show stepwise loss of Swc7 then Bdf1, consistent with our proposed interaction model. The interactions with Yaf9 also yield a consistent pattern of subunit deletion with clean loss of Yaf9 and Bdf1 but stable retention of all other subunits. Previous Yaf9 deletion studies using Western blots of complexes formed with the N-terminal region of Swr1 showed that, in addition to a total loss of Bdf1 and Yaf9, a partial loss of Swc4, actin, and Arp4 was also observed (27). Consequently, these observations produced a different interaction model from that we present in Figure 7. However, our data show clean losses or retention of subunits and are obtained in the context of larger Swr1 fragments. Also, the truncations and subunit deletions are internally consistent with one another.

There are also some important additional conclusions that we can draw from our truncation studies. We can now narrow down the interaction sites considerably and reveal the order of interactions along the N-terminal portion of Swr1. In common with the deletion studies, the more highly truncated complexes, that lose many subunits, lose the ability to couple ATP hydrolysis to histone exchange. Furthermore, the complex contains two actin subunits that appear to be located adjacent to one another along the Swr1 linear sequence. One of these subunits shows canonical interactions with Arp4 and the HSA region of Swr1 as seen for a variety of other related complexes that contain actin and Arp4 such as Ino80 and NuA4 (4,29). The crystal structure of a complex of actin, Arp4 and the HSA region of Swr1 (29) revealed the interaction sites on Swr1 for actin and Arp4. The Arp4 binding site spans Swr1 residues 355–367 and the actin binding site covers residues 373–388. These sites match very nicely with the N-terminal truncation experiments presented above (residues 370–409). The Arp4 subunit is lost completely in truncations beyond residue 370 (Supplementary Figure S1) and the first actin is lost after truncating beyond residue 410. However, a Swr1 protein that begins at residue 410 should have lost the entire region shown to contact actin in the crystal structure yet we show that it still binds actin. Truncation up to residue 460 then results in the loss of this second actin subunit suggesting the binding site is located between Swr1 residues 410 and 459. However, although the second subunit binds to this region, it also interacts with the Swc5 subunit and either or both are involved in interactions with the C-terminal region of the Swr1 subunit (Supplementary Figures S1 and S2). The selective deletion of these two actin binding sites individually and together (Supplementary Figure S2) confirm the existence of two bound actin molecules, each with distinct binding sites and interactions. This is different to other related complexes such as Ino80 (14,32) that contain a single actin subunit.

In addition to confirming interactions between an actin subunit and Swc5, suggested by our N-terminal truncations and the deletion complexes, our C-terminal truncations also reveal a role for this region of Swr1 in coupling nucleosome binding to ATPase and histone exchange activities. The loss of just 17 residues at the C-terminus of the Swr1 subunit ablates exchange activity without affecting ATPase activity. Consequently, the ATPase activity is uncoupled from nucleosome binding, most likely due to loss of the Swc5 subunit. However, further truncation, despite having no effect on subunit composition of the complex, actually stimulates ATPase and uncouples nucleosome binding from ATPase stimulation demonstrating a repression of ATPase that is mediated by the C-terminal region of Swr1. Interestingly, previous work has shown that deletion of a region near the C-terminus, but still retaining the last 50 or so residues of the protein, binds all subunits and is active in histone exchange (26). This is consistent with our data.

Previous structural studies of SWR1 have revealed a number of important features (10). First, the SWR1 complex contains a single heterohexamer of the Rvb proteins. This has been an issue of contention since crystal structures of the Rvb1/2 complex suggested a dodecamer was formed (22). An EM structure of SWR1 (10) was obtained by crosslinking with glutaraldehyde which was required to stabilise the complex so that it was sufficiently homogeneous for data collection. We decided not to crosslink the sample in case this stabilised a single state over others that are normally present. We used recent advances in EM single particle processing in RELION (19) to divide the sample into a variety of conformational states revealing considerable conformational heterogeneity that precluded a higher resolution analysis (Supplementary Figures S5 and S6). Similar observations have been reported by others (10,30). However, we were able to select groups of particles, each representing ∼20% of the total, that were sufficiently similar in conformation to allow 3D structures to be determined. The best class of our particles looked most similar to the crosslinked structure reported previously ((9) and Supplementary Figure S8) but others were more extended in conformation. Our best class allowed a structure determination at 21 Å resolution that shows more detail than previously reported structures at 28 Å resolution or less (10,30). Our structure suggests that there is actually a cavity within the complex that becomes closed after crosslinking and is therefore not evident in low resolution studies on the crosslinked complex. Together, our ensemble of five structures reveal a dynamic complex, with a continuum of conformational states between our two extreme classes (Supplementary Figure S6). It is quite possible that the conformation of the complex may be stabilised by the binding of nucleosomes, but further structural analysis of complexes with bound a bound nucleosome and/or histone dimer will be required to address that.

## Supplementary Material

Supplementary Data
