# Supplementary material for: Functional characterization and architecture of recombinant yeast SWR1 histone exchange complex
Source: Nucleic Acids Res. 2017 May 12;45(12):7249–60. doi: 10.1093/nar/gkx414 (PMC5499540; doi:10.1093/nar/gkx414)
Supplement: Supplementary Data [file gkx414_Supp.pdf]

Supplementary Material

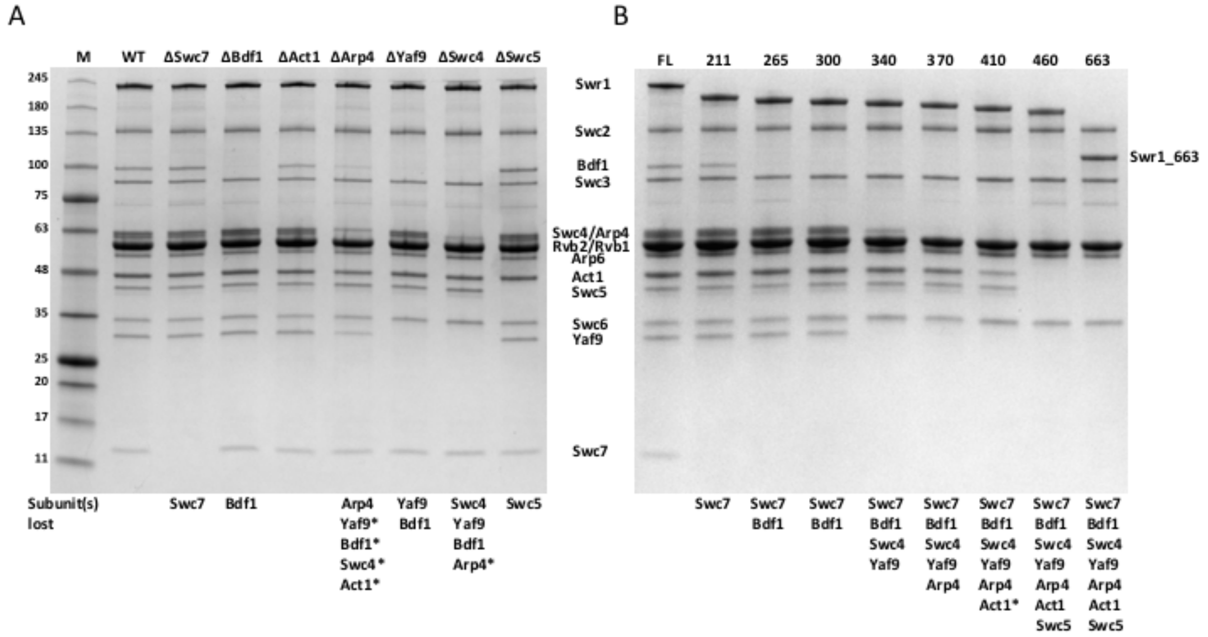

Supplementary Figure S1 – SDS gel of SWR1 complexes used in this study.

A) Subunit deletion complexes.

B) N-terminal truncation complexes.

Subunit identities are as marked. Asterisk indicates partial loss of subunit.

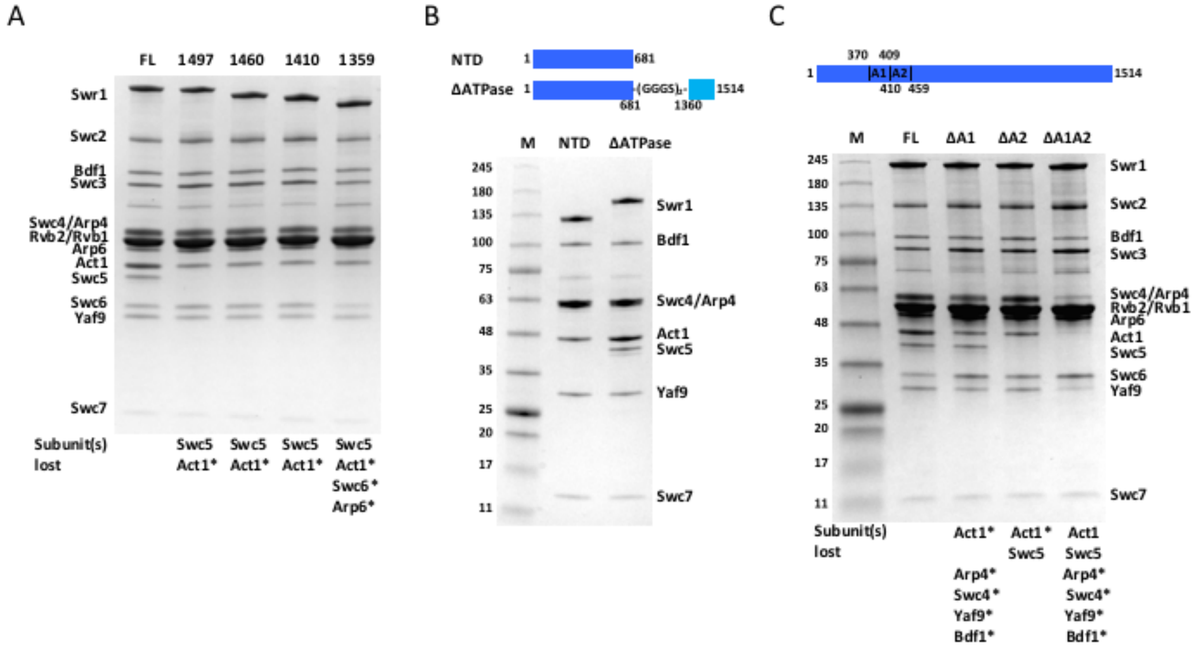

Supplementary Figure S2 – SDS gel of SWR1 complexes.

A) C-terminal truncation complexes.

B) NTD and ATPase complexes.

C) Internal deletion complexes in the HSA domain.

Identities of missing bands are as marked. Asterisk indicates partial loss of subunit.

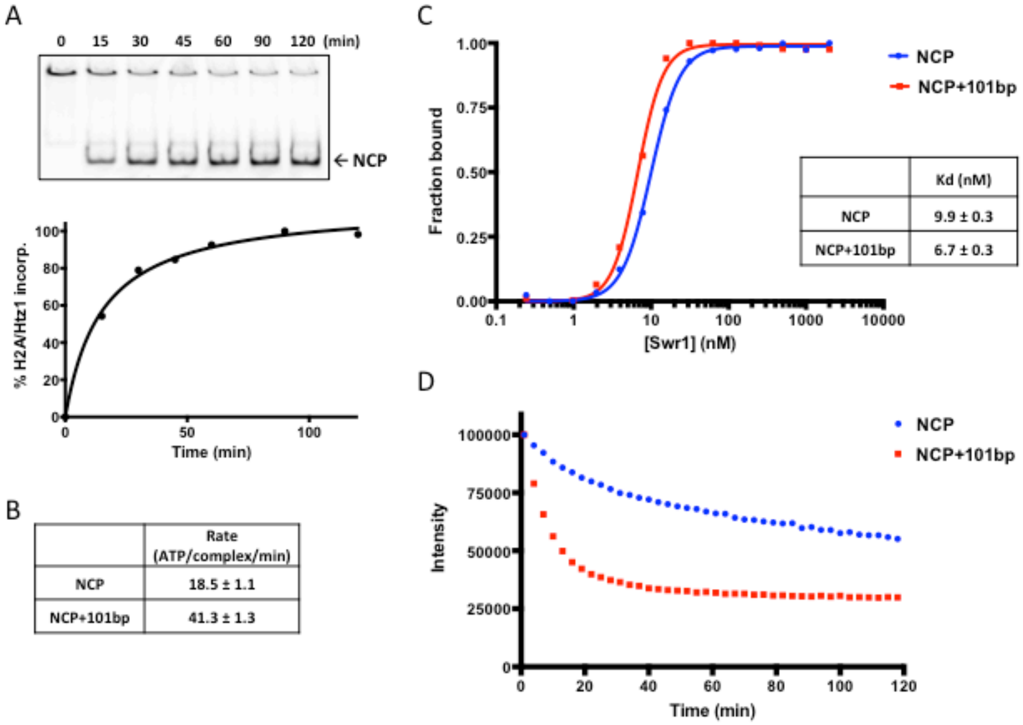

Supplementary Figure S3

A) Top - time-course gel-based histone exchange assay. Bottom - H2A/Htz1 incorporation curves based on a scan of the gel above.

B-D) ATPase rates, nucleosome binding affinities, histone exchange activity of SWR1 complex in the presence of yH2A nucleosome without or with 101bp overhang.

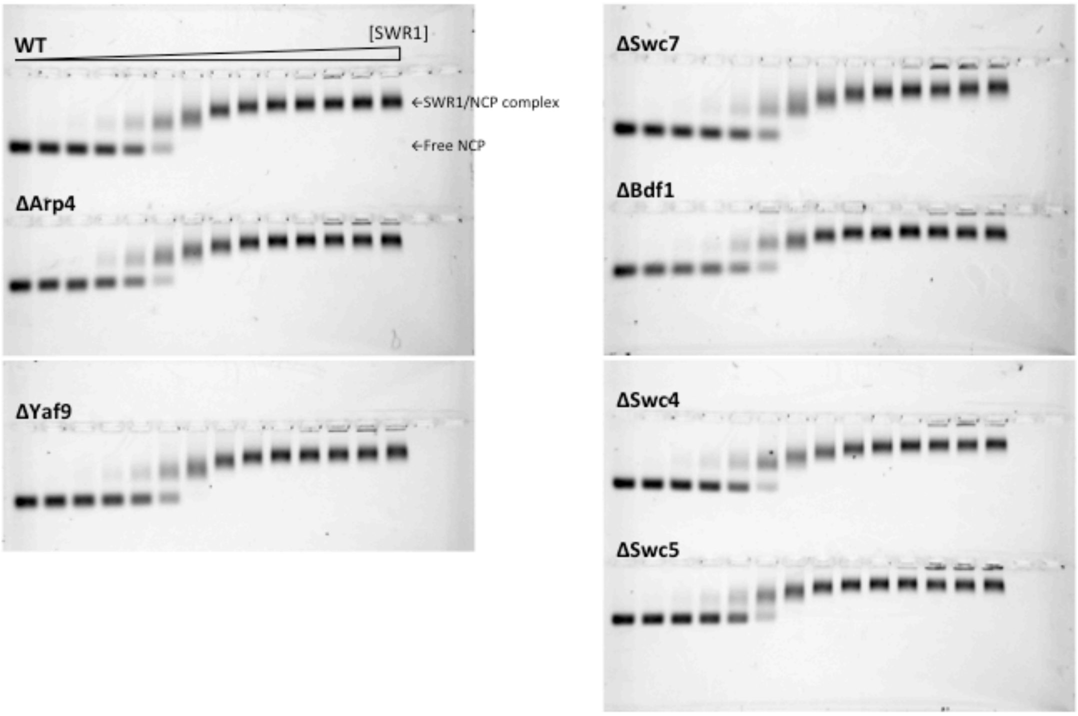

Supplementary Figure S4

Original native agarose gel images used to determine Kd values for wildtype and subunit deletion SWR1 complexes presented in Figure 2.

A

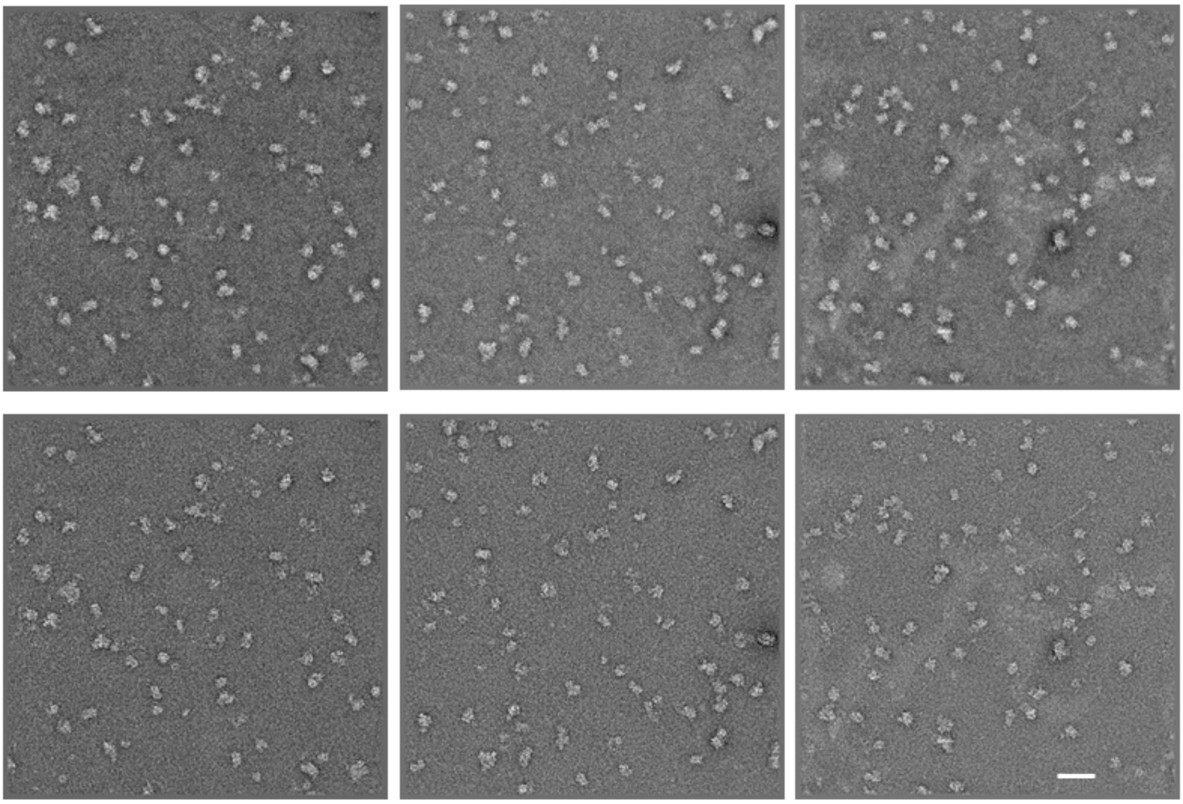

B

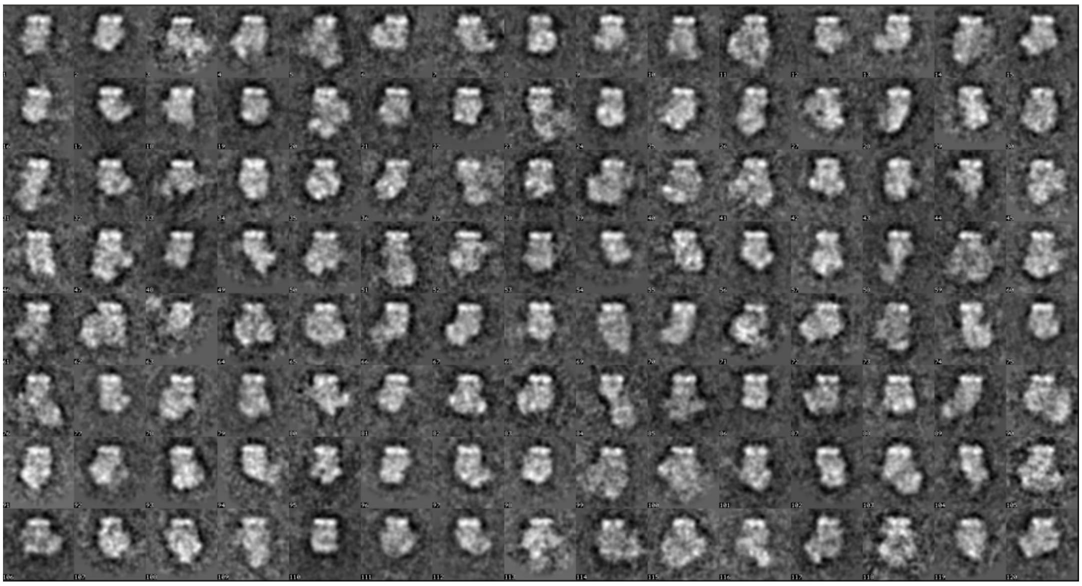

Supplementary Figure S5

A) Representative micrographs of the SWR1 sample. Top row shows raw single exposure micrographs. Bottom row shows sums of 3 consecutive exposures of the same area after amplitude normalisation. Space bar is 50 nm.

B) Classes representing side views of SWR1 complex were aligned based on the Rvb1/2 density (bright line at the top of the class). Despite this alignment, the lengths of the classes are clearly very different.

A

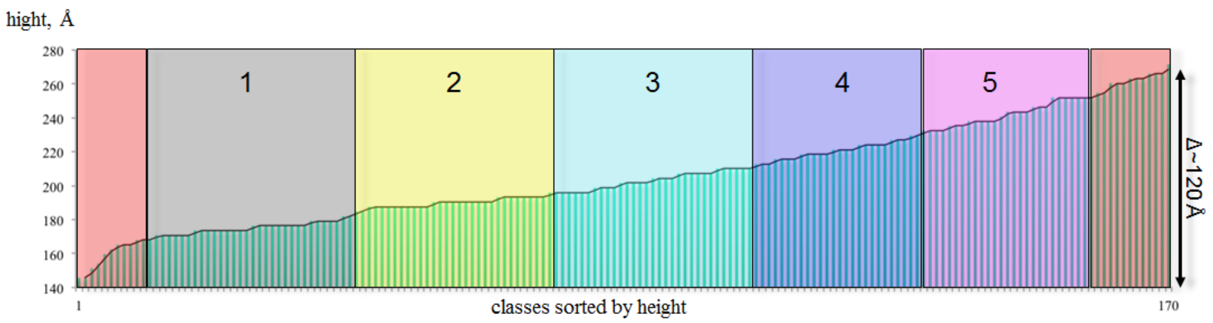

B

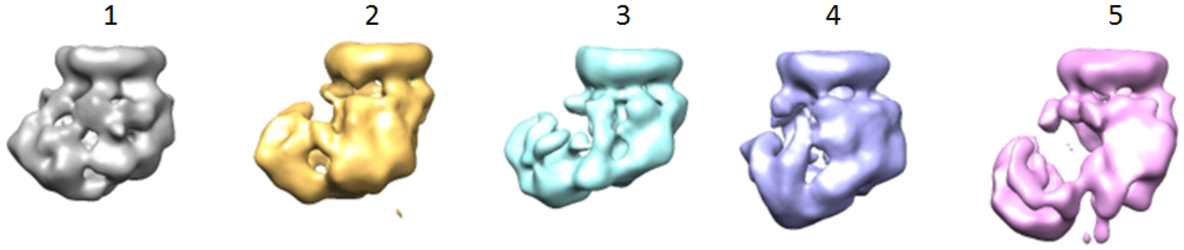

Supplementary Figure S6

A) Distribution of lengths of SWR1 side views. Particles contributing to the shortest and the longest classes were removed (indicated in red). The remaining particles were divided into five groups and subjected to further analysis.
B) Models of SWR1 after 3D classification and refinement in RELION with side view dimensions corresponding to the five length groups, coloured as in (A).

A

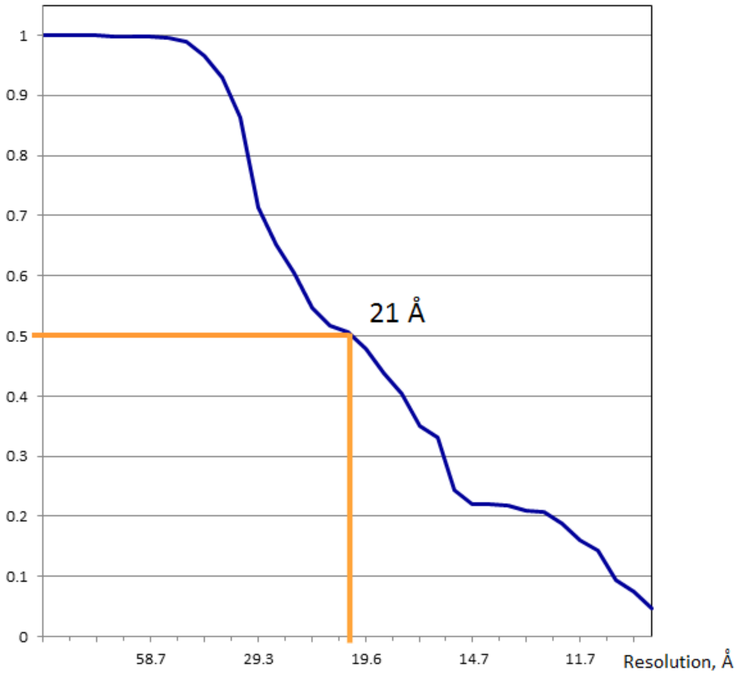

B

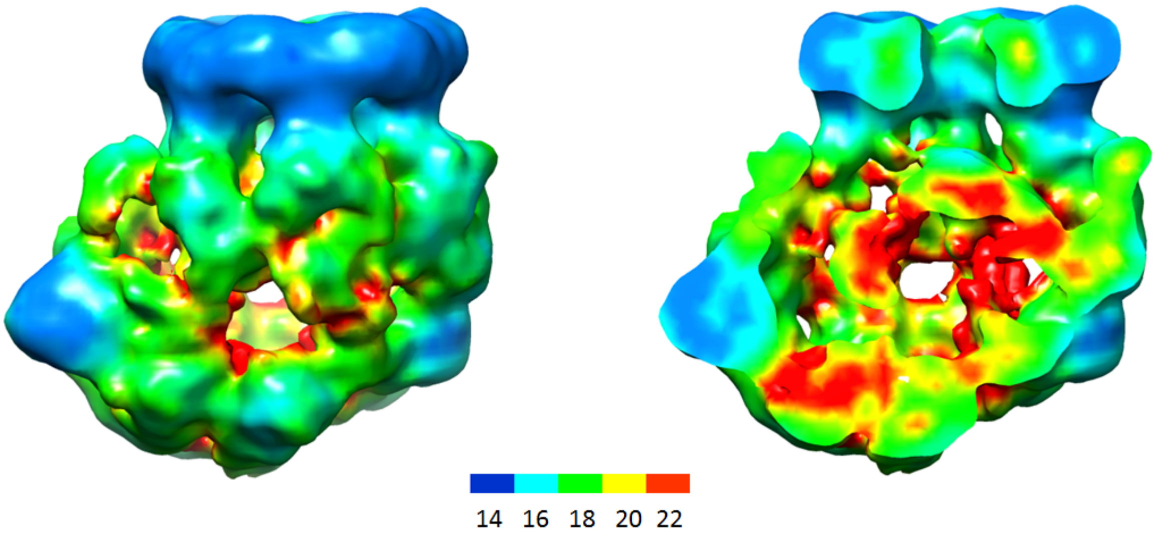

Supplementary Figure S7

A) Fourier Shell Correlation of the most compact SWR1 model. Resolution was estimated from the 0.5 cut-off criterion.

B) Local resolution estimation. Left panel - surface view, right panel - cut through to show the resolution distribution inside. Colour scale bar shows resolution in Å.

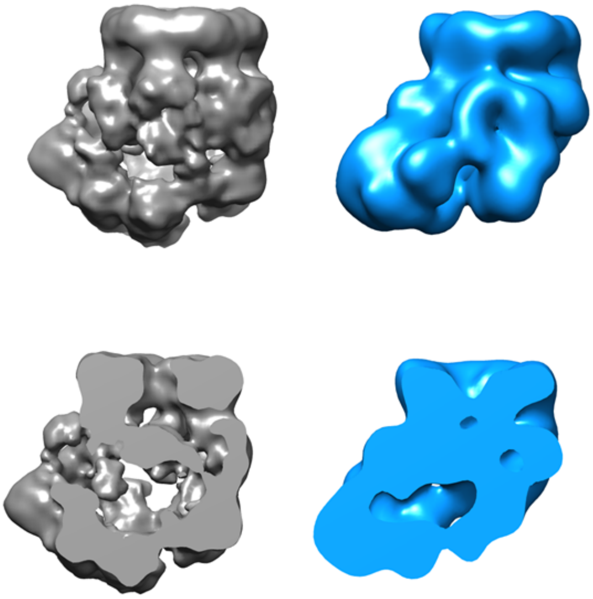

Supplementary Figure S8

Comparison of our most compact model of SWR1 (grey) with the previously reported cross-linked model of SWR1 complex (blue, Nguyen et al., 2013; EMDB 5626). The overall dimensions of the models are similar, however our model exhibits greater structural detail and contains a larger central cavity. Top - surface view. Bottom - cut away view.
